# *n*-Butyl­dichlorido(2-{(1*E*)-1-[2-(pyridin-2-yl)hydrazin-1-yl­idene]eth­yl}phenolato)tin(IV)

**DOI:** 10.1107/S1600536810040572

**Published:** 2010-10-20

**Authors:** Md. Abu Affan, Dayang N. A. Chee, Fasihuddin B. Ahmad, Seik Weng Ng, Edward R. T. Tiekink

**Affiliations:** a Faculty of Resource Science and Technology, Universiti Malaysia Sarawak, 94300 Kota Samarahan, Sarawak, Malaysia; b Department of Chemistry, University of Malaya, 50603 Kuala Lumpur, Malaysia

## Abstract

Two independent mol­ecules comprise the asymmetric unit of the title compound, [Sn(C_4_H_9_)(C_13_H_12_N_3_O)Cl_2_]. The Sn atom in each is coordinated by the tridentate ligand *via* the phenoxide O, hydrazine N and pyridyl N atoms, forming five- and six-membered chelate rings. The approximately octa­hedral coordination geometry is completed by the α-C atom of the *n*-butyl group (which is *trans* to the hydrazine N atom) and two mutually *trans* Cl atoms. Differences between the mol­ecules are evident in the relative planarity of the chelate rings and in the conformations of the *n*-butyl groups [C—C—C—C = 177.2 (5) and −64.4 (11)°]. Significant differences in the Sn—Cl bond lengths are related to the formation of N—H⋯Cl hydrogen bonds, which link the mol­ecules comprising the asymmetric unit into dimeric aggregates. These are consolidated in the crystal packing by C—H⋯Cl contacts. The structure was refined as an inversion twin; the minor twin component is 37 (3)%.

## Related literature

For background to related organotin compounds, see: Affan *et al.* (2009 ▶). For background to the varied biological activities of organotin compounds, see: Gielen & Tiekink (2005 ▶). For additional structure analysis, see: Spek (2009 ▶).
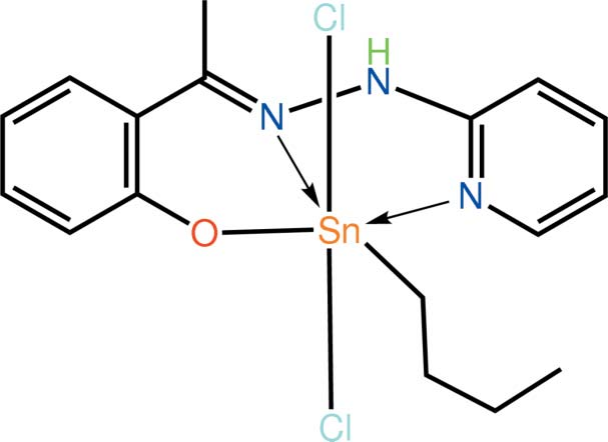


## Experimental

### 

#### Crystal data

[Sn(C_4_H_9_)(C_13_H_12_N_3_O)Cl_2_]
*M* *_r_* = 472.96
Monoclinic, 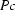

*a* = 8.9566 (6) Å
*b* = 21.0210 (13) Å
*c* = 10.3974 (7) Å
β = 110.567 (1)°
*V* = 1832.8 (2) Å^3^
*Z* = 4
Mo *K*α radiation
μ = 1.70 mm^−1^
*T* = 100 K
0.25 × 0.15 × 0.05 mm

#### Data collection

Bruker SMART APEX CCD diffractometer
Absorption correction: multi-scan (*SADABS*; Sheldrick, 1996 ▶) *T*_min_ = 0.677, *T*_max_ = 0.920
17203 measured reflections
8239 independent reflections
7357 reflections with *I* > 2σ(*I*)
*R*_int_ = 0.032

#### Refinement

*R*[*F* ^2^ > 2σ(*F* ^2^)] = 0.042
*wR*(*F* ^2^) = 0.108
*S* = 1.02
8239 reflections
442 parameters
4 restraints
H atoms treated by a mixture of independent and constrained refinement
Δρ_max_ = 2.28 e Å^−3^
Δρ_min_ = −1.21 e Å^−3^
Absolute structure: Flack (1983 ▶), 4006 Friedel pairs
Flack parameter: 0.37 (3)

### 

Data collection: *APEX2* (Bruker, 2008 ▶); cell refinement: *SAINT* (Bruker, 2008 ▶); data reduction: *SAINT*; program(s) used to solve structure: *SHELXS97* (Sheldrick, 2008 ▶); program(s) used to refine structure: *SHELXL97* (Sheldrick, 2008 ▶); molecular graphics: *ORTEP-3* (Farrugia, 1997 ▶) and *DIAMOND* (Brandenburg, 2006 ▶); software used to prepare material for publication: *PLATON* (Spek, 2009 ▶) and *publCIF* (Westrip, 2010 ▶).

## Supplementary Material

Crystal structure: contains datablocks global, I. DOI: 10.1107/S1600536810040572/hb5679sup1.cif

Structure factors: contains datablocks I. DOI: 10.1107/S1600536810040572/hb5679Isup2.hkl

Additional supplementary materials:  crystallographic information; 3D view; checkCIF report

## Figures and Tables

**Table 1:** Selected bond lengths (Å)

Sn1—Cl1	2.4504 (17)
Sn1—Cl2	2.5225 (16)
Sn1—O1	2.004 (5)
Sn1—N1	2.266 (6)
Sn1—N3	2.198 (6)
Sn1—C4	2.169 (7)
Sn2—Cl3	2.456 (2)
Sn2—Cl4	2.5116 (18)
Sn2—O2	2.017 (5)
Sn2—N4	2.248 (6)
Sn2—N6	2.212 (7)
Sn2—C21	2.142 (7)

**Table 2:** Hydrogen-bond geometry (Å, °)

*D*—H⋯*A*	*D*—H	H⋯*A*	*D*⋯*A*	*D*—H⋯*A*
N2—H2n⋯Cl4	0.86 (3)	2.47 (4)	3.283 (7)	159 (7)
N5—H5n⋯Cl2	0.86 (3)	2.48 (5)	3.235 (7)	147 (7)
C15—H15⋯Cl2^i^	0.95	2.79	3.538 (9)	137
C17—H17⋯Cl1^ii^	0.95	2.80	3.540 (9)	135
C34—H34⋯Cl3^iii^	0.95	2.75	3.534 (9)	141

Symmetry codes: (i) 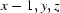; (ii) 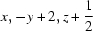; (iii) 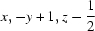.

**Fig. 1.**
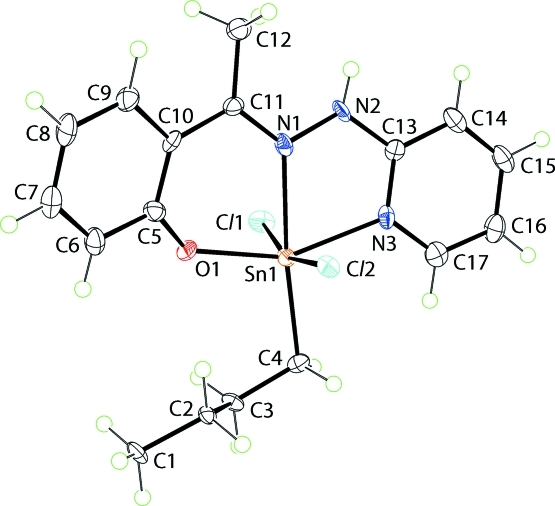
The molecular structure of the first independent molecule in (I) showing displacement ellipsoids at the 50% probability level.

**Fig. 2.**
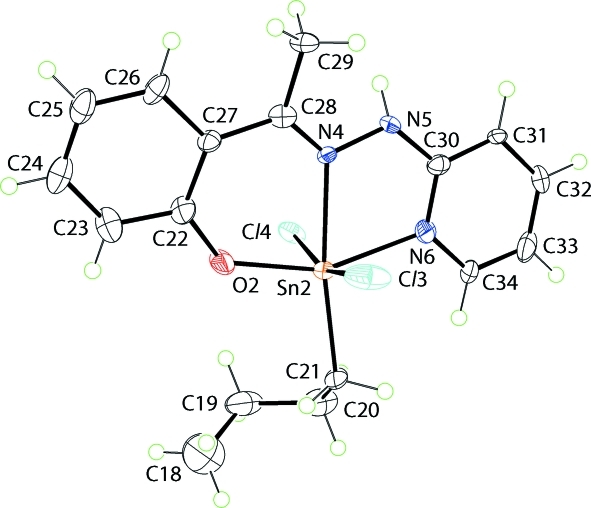
The molecular structure of the second independent molecule in (I) showing displacement ellipsoids at the 50% probability level.

**Fig. 3.**
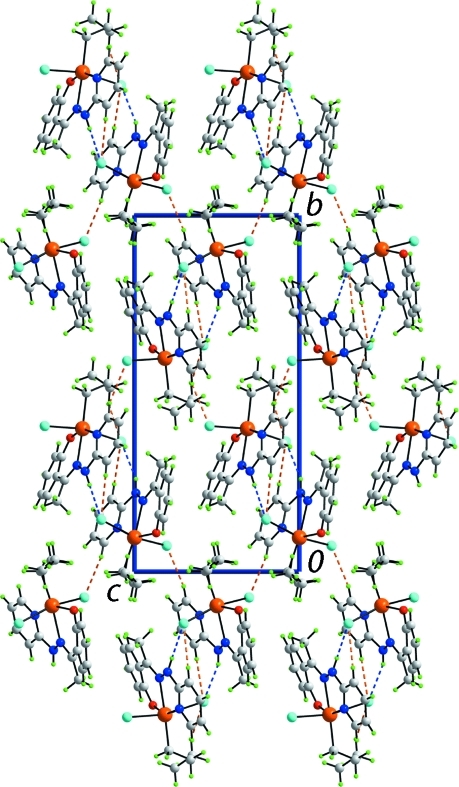
Unit-cell contents shown in projection down the a axis in (I). The N–H···Cl hydrogen bonding and C–H···Cl contacts are shown as blue and orange dashed lines, respectively.

## supplementary crystallographic information

### Comment

The title compound was examined in connection with synthetic studies of organotin(IV) compounds with pyruvic acid-2-pyridylhydrazone ligands (Affan *et al.*, 2009), studies motivated by their putative biological activity (Gielen & Tiekink, 2005).

Two independent molecules comprise the asymmetric unit of (I), Figs 1 and 2. The Sn atom in each is coordinated by the tridentate ligand *via* the phenoxide-O, hydrazine-N and pyridyl-N atoms to form five- and six-membered chelate rings, Table 1. For the Sn1 atom, distortions of these rings from planarity [r.m.s. deviation from the five- and six-membered rings = 0.083 and 0.216 Å, respectively] are greater than the equivalent rings involving the Sn2 atom [r.m.s. = 0.042 and 0.109 Å, respectively]. The dihedral angle formed between the chelate rings = 8.9 (3) ° for Sn1, and 7.4 (3) ° for Sn2. Overall, the tridentate ligand deviates further from co-planarity for the Sn1 atom compared to the Sn2 atom as seen in the respective dihedral angles formed between the pyridyl and benzene rings of 23.3 (4) and 17.3 (4) °. The coordination geometry is completed by two chlorido atoms and the alpha-C atom of the *n*-butyl group. The chlorido atoms occupy mutually *trans* positions and the butyl-C atom is *trans* to the hydrazine-N atom. The resulting CCl_2_N_2_O donor set defines an approximate octahedron. A further difference between the independent molecules is found in the conformation of the *n*-butyl groups. This is reflected in the C1—C2—C3—C4 and C2—C3—C4—Sn1 torsion angles of 177.2 (5) and -71.1 (6) °, respectively, compared to the C18—C19—C20—C21 and C19—C20—C21—Sn2 angles of -64.4 (11) and -72.9 (9) °, respectively.

As noted in Table 1, there are disparities in the Sn—Cl bond distances. This is directly related to the participation of the Cl2 and Cl4 atoms in N—H···Cl hydrogen bonding interactions, Table2, which connect the molecules comprising the asymmetric unit into dimeric aggregates. The latter are sustained in the crystal packing by C—H···Cl contacts, Fig. 3 and Table 2.

### Experimental

2-Hydroxyacetophenone 2-pyridylhydrazone (0.45 g, 0.002 mol) was dissolved in hot absolute methanol (20 ml) in a Schlenk round bottom flask under an nitrogen atmosphere. Potassium hydroxide (0.11 g, 0.002 mol) dissolved in methanol (5 ml) was added drop wise to the solution, resulting in a colour change from yellow to orange. The resulting mixture was refluxed for 1 h and a solution of *n*-BuSnCl_3_ (0.56 g, 0.002 mol) in methanol (10 ml) was added drop wise to the refluxed solution. The resulting mixture was refluxed for 5 h and allowed to cool to room temperature. The precipitated KCl was removed *via* filtration. Then the filtrate was evaporated to dryness by using a rotary evaporator to obtain orange microcrystals. The orange microcrystals were filtered off, washed with cold methanol and dried overnight over P_2_O_5_*in vacuo*. Single crystals of (I) were obtained by slow evaporation of its methanol solution at room temperature. Yield: 0.67 g, 60%. *M*.pt. 538–540 K.

### Refinement

Carbon-bound H-atoms were placed in calculated positions (C–H = 0.95 to 0.99 Å) and were included in the refinement in the riding model approximation, with *U*_iso_(H) set to 1.2–1.5*U*_equiv_(C). The N-bound H-atoms were located in a difference Fourier map and were refined with a distance restraint of N—H 0.86±0.01 Å, and with *U*_iso_(H) = 1.2*U*_eq_(N). The maximum and minimum residual electron density peaks of 2.28 and 1.21 e Å^-3^, respectively, were located 1.16 Å and 0.76 Å from the Sn2 and Cl3 atoms, respectively. The structure was refined as an inversion twin. The use of this twin law gave the minor twin component as 37 (3)%.

### Crystal data

**Table:**

[Sn(C_4_H_9_)(C_13_H_12_N_3_O)Cl_2_]	*F*(000) = 944
*M**_r_* = 472.96	*D*_x_ = 1.714 Mg m^−^^3^
Monoclinic, *P**c*	Mo *K*α radiation, λ = 0.71073 Å
Hall symbol: P -2yc	Cell parameters from 6860 reflections
*a* = 8.9566 (6) Å	θ = 2.3–28.2°
*b* = 21.0210 (13) Å	µ = 1.70 mm^−^^1^
*c* = 10.3974 (7) Å	*T* = 100 K
β = 110.567 (1)°	Prism, orange
*V* = 1832.8 (2) Å^3^	0.25 × 0.15 × 0.05 mm
*Z* = 4	

### Data collection

**Table:**

Bruker SMART APEX CCD diffractometer	8239 independent reflections
Radiation source: fine-focus sealed tube	7357 reflections with *I* > 2σ(*I*)
graphite	*R*_int_ = 0.032
ω scans	θ_max_ = 27.5°, θ_min_ = 1.0°
Absorption correction: multi-scan (*SADABS*; Sheldrick, 1996)	*h* = −11→11
*T*_min_ = 0.677, *T*_max_ = 0.920	*k* = −26→27
17203 measured reflections	*l* = −13→13

### Refinement

**Table:**

Refinement on *F*^2^	Secondary atom site location: difference Fourier map
Least-squares matrix: full	Hydrogen site location: inferred from neighbouring sites
*R*[*F*^2^ > 2σ(*F*^2^)] = 0.042	H atoms treated by a mixture of independent and constrained refinement
*wR*(*F*^2^) = 0.108	*w* = 1/[σ^2^(*F*_o_^2^) + (0.0471*P*)^2^ + 7.3356*P*] where *P* = (*F*_o_^2^ + 2*F*_c_^2^)/3
*S* = 1.02	(Δ/σ)_max_ = 0.001
8239 reflections	Δρ_max_ = 2.28 e Å^−^^3^
442 parameters	Δρ_min_ = −1.21 e Å^−^^3^
4 restraints	Absolute structure: Flack (1983), 4006 Friedel pairs
Primary atom site location: structure-invariant direct methods	Flack parameter: 0.37 (3)

### Special details

**Table:**

Geometry. All s.u.'s (except the s.u. in the dihedral angle between two l.s. planes) are estimated using the full covariance matrix. The cell s.u.'s are taken into account individually in the estimation of s.u.'s in distances, angles and torsion angles; correlations between s.u.'s in cell parameters are only used when they are defined by crystal symmetry. An approximate (isotropic) treatment of cell s.u.'s is used for estimating s.u.'s involving l.s. planes.
Refinement. Refinement of *F*^2^ against ALL reflections. The weighted *R*-factor *wR* and goodness of fit *S* are based on *F*^2^, conventional *R*-factors *R* are based on *F*, with *F* set to zero for negative *F*^2^. The threshold expression of *F*^2^ > 2σ(*F*^2^) is used only for calculating *R*-factors(gt) *etc*. and is not relevant to the choice of reflections for refinement. *R*-factors based on *F*^2^ are statistically about twice as large as those based on *F*, and *R*- factors based on ALL data will be even larger.

### Fractional atomic coordinates and isotropic or equivalent isotropic displacement parameters (Å^2^)

**Table:**

	*x*	*y*	*z*	*U*_iso_*/*U*_eq_	
Sn1	0.49996 (4)	0.90443 (2)	0.50000 (3)	0.01474 (11)	
Sn2	0.43258 (4)	0.59873 (2)	0.81542 (4)	0.01729 (12)	
Cl1	0.2560 (2)	0.92741 (8)	0.30505 (18)	0.0234 (4)	
Cl2	0.71385 (18)	0.85448 (8)	0.70222 (17)	0.0170 (3)	
Cl3	0.6382 (3)	0.59138 (10)	1.0451 (2)	0.0429 (6)	
Cl4	0.2538 (2)	0.63490 (9)	0.58185 (19)	0.0263 (4)	
O1	0.6157 (6)	0.8917 (2)	0.3683 (5)	0.0195 (10)	
O2	0.2612 (7)	0.6248 (3)	0.8890 (6)	0.0351 (14)	
N1	0.4371 (7)	0.8008 (3)	0.4495 (7)	0.0174 (13)	
N2	0.3150 (8)	0.7796 (3)	0.4922 (7)	0.0182 (13)	
H2N	0.289 (9)	0.7402 (11)	0.492 (8)	0.022*	
N3	0.3350 (8)	0.8773 (3)	0.6049 (6)	0.0177 (13)	
N4	0.5039 (7)	0.7016 (3)	0.8490 (7)	0.0159 (12)	
N5	0.6249 (8)	0.7180 (3)	0.7999 (7)	0.0172 (13)	
H5N	0.607 (9)	0.7568 (12)	0.773 (7)	0.021*	
N6	0.6218 (8)	0.6147 (3)	0.7280 (8)	0.0233 (14)	
C1	0.9471 (7)	1.0332 (3)	0.4834 (6)	0.0184 (13)	
H1A	1.0541	1.0145	0.5185	0.028*	
H1B	0.9545	1.0792	0.4993	0.028*	
H1C	0.8986	1.0247	0.3847	0.028*	
C2	0.8449 (7)	1.0038 (3)	0.5573 (6)	0.0138 (11)	
H2A	0.8947	1.0121	0.6571	0.017*	
H2B	0.8403	0.9572	0.5433	0.017*	
C3	0.6756 (7)	1.0307 (3)	0.5055 (6)	0.0146 (12)	
H3A	0.6813	1.0771	0.5233	0.018*	
H3B	0.6294	1.0245	0.4048	0.018*	
C4	0.5633 (8)	1.0012 (3)	0.5702 (7)	0.0170 (14)	
H4A	0.4652	1.0273	0.5465	0.020*	
H4B	0.6153	1.0014	0.6714	0.020*	
C5	0.6916 (9)	0.8399 (3)	0.3502 (7)	0.0185 (15)	
C6	0.8343 (9)	0.8498 (4)	0.3249 (7)	0.0221 (15)	
H6	0.8745	0.8918	0.3275	0.027*	
C7	0.9166 (9)	0.7998 (4)	0.2965 (7)	0.0244 (15)	
H7	1.0135	0.8077	0.2813	0.029*	
C8	0.8605 (9)	0.7386 (4)	0.2896 (8)	0.0272 (17)	
H8	0.9174	0.7045	0.2684	0.033*	
C9	0.7194 (9)	0.7269 (3)	0.3140 (7)	0.0222 (15)	
H9	0.6801	0.6847	0.3091	0.027*	
C10	0.6336 (8)	0.7781 (3)	0.3465 (7)	0.0150 (14)	
C11	0.4906 (9)	0.7603 (3)	0.3786 (7)	0.0164 (15)	
C12	0.4144 (10)	0.6970 (3)	0.3391 (8)	0.0225 (16)	
H12A	0.2983	0.7015	0.3095	0.034*	
H12B	0.4517	0.6682	0.4181	0.034*	
H12C	0.4431	0.6795	0.2635	0.034*	
C13	0.2705 (9)	0.8186 (4)	0.5790 (8)	0.0165 (14)	
C14	0.1616 (10)	0.7971 (4)	0.6375 (8)	0.0248 (17)	
H14	0.1227	0.7547	0.6234	0.030*	
C15	0.1121 (9)	0.8383 (4)	0.7152 (8)	0.0252 (17)	
H15	0.0328	0.8254	0.7509	0.030*	
C16	0.1784 (10)	0.9006 (4)	0.7434 (9)	0.0259 (18)	
H16	0.1478	0.9293	0.8003	0.031*	
C17	0.2872 (9)	0.9172 (4)	0.6856 (8)	0.0224 (16)	
H17	0.3320	0.9587	0.7024	0.027*	
C18	0.0622 (13)	0.4490 (6)	0.7565 (13)	0.069 (3)	
H18A	−0.0502	0.4577	0.7416	0.103*	
H18B	0.1286	0.4637	0.8482	0.103*	
H18C	0.0772	0.4031	0.7492	0.103*	
C19	0.1096 (11)	0.4834 (4)	0.6495 (10)	0.045 (2)	
H19A	0.0931	0.5296	0.6574	0.054*	
H19B	0.0384	0.4697	0.5573	0.054*	
C20	0.2852 (11)	0.4721 (4)	0.6612 (9)	0.042 (2)	
H20A	0.3035	0.4259	0.6562	0.050*	
H20B	0.3038	0.4927	0.5825	0.050*	
C21	0.4014 (9)	0.4978 (3)	0.7918 (9)	0.0265 (18)	
H21A	0.5068	0.4789	0.8043	0.032*	
H21B	0.3693	0.4821	0.8680	0.032*	
C22	0.2329 (10)	0.6778 (4)	0.9437 (8)	0.0244 (17)	
C23	0.0932 (10)	0.6776 (4)	0.9770 (8)	0.0301 (18)	
H23	0.0294	0.6403	0.9601	0.036*	
C24	0.0472 (10)	0.7291 (5)	1.0322 (8)	0.0325 (19)	
H24	−0.0489	0.7279	1.0515	0.039*	
C25	0.1403 (11)	0.7833 (4)	1.0605 (8)	0.0345 (19)	
H25	0.1090	0.8190	1.1012	0.041*	
C26	0.2780 (10)	0.7861 (4)	1.0302 (7)	0.0266 (17)	
H26	0.3400	0.8239	1.0503	0.032*	
C27	0.3303 (9)	0.7333 (3)	0.9691 (8)	0.0188 (15)	
C28	0.4673 (10)	0.7429 (4)	0.9257 (7)	0.0200 (16)	
C29	0.5648 (9)	0.8019 (3)	0.9702 (8)	0.0230 (16)	
H29A	0.6765	0.7925	0.9835	0.034*	
H29B	0.5572	0.8171	1.0568	0.034*	
H29C	0.5248	0.8348	0.8996	0.034*	
C30	0.6775 (9)	0.6730 (3)	0.7290 (8)	0.0186 (15)	
C31	0.7888 (9)	0.6884 (3)	0.6693 (8)	0.0174 (14)	
H31	0.8311	0.7302	0.6753	0.021*	
C32	0.8356 (9)	0.6406 (4)	0.6005 (8)	0.0236 (16)	
H32	0.9098	0.6496	0.5565	0.028*	
C33	0.7758 (10)	0.5798 (4)	0.5953 (10)	0.0321 (19)	
H33	0.8093	0.5467	0.5493	0.039*	
C34	0.6673 (9)	0.5683 (4)	0.6575 (9)	0.0251 (17)	
H34	0.6224	0.5269	0.6517	0.030*	

### Atomic displacement parameters (Å^2^)

**Table:**

	*U*^11^	*U*^22^	*U*^33^	*U*^12^	*U*^13^	*U*^23^
Sn1	0.0153 (2)	0.0133 (2)	0.0172 (2)	0.00001 (19)	0.00764 (19)	0.00070 (19)
Sn2	0.0182 (2)	0.0132 (2)	0.0207 (2)	−0.00161 (19)	0.0071 (2)	−0.00055 (19)
Cl1	0.0231 (8)	0.0194 (8)	0.0223 (8)	0.0007 (6)	0.0014 (7)	0.0046 (6)
Cl2	0.0148 (7)	0.0153 (7)	0.0211 (7)	0.0007 (6)	0.0065 (6)	0.0025 (6)
Cl3	0.0488 (13)	0.0265 (10)	0.0326 (11)	−0.0111 (9)	−0.0119 (9)	0.0101 (8)
Cl4	0.0220 (8)	0.0242 (9)	0.0278 (9)	−0.0064 (7)	0.0026 (7)	0.0074 (7)
O1	0.026 (3)	0.013 (2)	0.023 (2)	0.0013 (19)	0.014 (2)	−0.0039 (18)
O2	0.038 (3)	0.024 (3)	0.056 (4)	−0.016 (3)	0.033 (3)	−0.022 (3)
N1	0.012 (3)	0.023 (3)	0.020 (3)	−0.002 (2)	0.009 (2)	0.001 (2)
N2	0.016 (3)	0.021 (3)	0.021 (3)	−0.006 (2)	0.011 (2)	0.003 (2)
N3	0.020 (3)	0.020 (3)	0.018 (3)	0.004 (2)	0.012 (2)	0.003 (2)
N4	0.013 (3)	0.010 (3)	0.024 (3)	0.001 (2)	0.005 (2)	0.000 (2)
N5	0.017 (3)	0.013 (3)	0.024 (3)	−0.003 (2)	0.009 (3)	0.002 (2)
N6	0.012 (3)	0.022 (3)	0.033 (4)	−0.002 (2)	0.003 (3)	−0.004 (3)
C1	0.012 (3)	0.024 (3)	0.019 (3)	−0.007 (2)	0.006 (2)	0.004 (3)
C2	0.017 (3)	0.014 (3)	0.011 (3)	−0.004 (2)	0.005 (2)	0.002 (2)
C3	0.014 (3)	0.010 (3)	0.019 (3)	0.000 (2)	0.004 (2)	0.005 (2)
C4	0.015 (3)	0.016 (3)	0.018 (3)	0.001 (3)	0.004 (3)	−0.004 (3)
C5	0.020 (3)	0.019 (3)	0.016 (3)	−0.004 (3)	0.005 (3)	0.000 (3)
C6	0.016 (3)	0.033 (4)	0.019 (3)	−0.001 (3)	0.008 (3)	−0.001 (3)
C7	0.019 (3)	0.038 (4)	0.017 (3)	0.004 (3)	0.008 (3)	0.001 (3)
C8	0.023 (4)	0.037 (5)	0.029 (4)	0.006 (3)	0.017 (3)	−0.003 (3)
C9	0.030 (4)	0.016 (3)	0.024 (4)	0.005 (3)	0.014 (3)	0.001 (3)
C10	0.014 (3)	0.019 (3)	0.013 (3)	0.003 (3)	0.005 (3)	−0.005 (2)
C11	0.023 (4)	0.012 (3)	0.014 (3)	0.000 (3)	0.006 (3)	0.000 (3)
C12	0.029 (4)	0.019 (3)	0.019 (3)	−0.002 (3)	0.007 (3)	−0.001 (3)
C13	0.010 (3)	0.023 (4)	0.014 (3)	−0.001 (3)	0.002 (3)	−0.002 (3)
C14	0.024 (4)	0.026 (4)	0.024 (4)	−0.004 (3)	0.009 (3)	0.008 (3)
C15	0.017 (3)	0.034 (4)	0.026 (4)	−0.004 (3)	0.009 (3)	0.008 (3)
C16	0.021 (4)	0.035 (5)	0.024 (4)	0.006 (3)	0.012 (3)	−0.001 (3)
C17	0.020 (4)	0.026 (4)	0.019 (3)	0.002 (3)	0.004 (3)	0.004 (3)
C18	0.044 (6)	0.076 (8)	0.069 (7)	0.009 (6)	−0.002 (5)	0.031 (6)
C19	0.042 (5)	0.025 (4)	0.055 (5)	−0.002 (4)	0.000 (4)	0.005 (4)
C20	0.045 (5)	0.030 (4)	0.043 (5)	0.000 (4)	0.006 (4)	0.001 (4)
C21	0.024 (4)	0.014 (3)	0.043 (5)	−0.003 (3)	0.015 (4)	−0.012 (3)
C22	0.027 (4)	0.028 (4)	0.019 (3)	−0.002 (3)	0.010 (3)	−0.009 (3)
C23	0.026 (4)	0.047 (5)	0.017 (3)	−0.006 (4)	0.008 (3)	−0.006 (3)
C24	0.030 (5)	0.045 (5)	0.023 (4)	0.014 (4)	0.009 (3)	−0.003 (4)
C25	0.041 (5)	0.039 (5)	0.031 (4)	0.016 (4)	0.023 (4)	0.009 (3)
C26	0.035 (4)	0.028 (4)	0.021 (4)	0.012 (3)	0.014 (3)	0.000 (3)
C27	0.019 (4)	0.017 (3)	0.019 (3)	−0.001 (3)	0.005 (3)	−0.005 (3)
C28	0.026 (4)	0.020 (4)	0.011 (3)	−0.001 (3)	0.003 (3)	0.001 (3)
C29	0.030 (4)	0.015 (3)	0.024 (4)	−0.005 (3)	0.009 (3)	−0.003 (3)
C30	0.019 (4)	0.016 (3)	0.018 (3)	0.002 (3)	0.003 (3)	0.003 (3)
C31	0.014 (3)	0.009 (3)	0.027 (4)	0.001 (2)	0.005 (3)	0.004 (3)
C32	0.015 (3)	0.033 (4)	0.026 (4)	−0.001 (3)	0.011 (3)	0.000 (3)
C33	0.016 (4)	0.037 (5)	0.040 (5)	0.007 (3)	0.005 (3)	−0.010 (4)
C34	0.019 (4)	0.020 (4)	0.041 (5)	0.002 (3)	0.016 (3)	−0.005 (3)

### Geometric parameters (Å, °)

**Table:**

Sn1—Cl1	2.4504 (17)	C10—C11	1.481 (10)
Sn1—Cl2	2.5225 (16)	C11—C12	1.486 (10)
Sn1—O1	2.004 (5)	C12—H12A	0.9800
Sn1—N1	2.266 (6)	C12—H12B	0.9800
Sn1—N3	2.198 (6)	C12—H12C	0.9800
Sn1—C4	2.169 (7)	C13—C14	1.394 (10)
Sn2—Cl3	2.456 (2)	C14—C15	1.359 (11)
Sn2—Cl4	2.5116 (18)	C14—H14	0.9500
Sn2—O2	2.017 (5)	C15—C16	1.426 (11)
Sn2—N4	2.248 (6)	C15—H15	0.9500
Sn2—N6	2.212 (7)	C16—C17	1.358 (11)
Sn2—C21	2.142 (7)	C16—H16	0.9500
O1—C5	1.332 (9)	C17—H17	0.9500
O2—C22	1.314 (9)	C18—C19	1.508 (15)
N1—C11	1.321 (9)	C18—H18A	0.9800
N1—N2	1.390 (8)	C18—H18B	0.9800
N2—C13	1.378 (10)	C18—H18C	0.9800
N2—H2n	0.86 (3)	C19—C20	1.553 (13)
N3—C13	1.349 (10)	C19—H19A	0.9900
N3—C17	1.358 (10)	C19—H19B	0.9900
N4—C28	1.295 (10)	C20—C21	1.492 (11)
N4—N5	1.394 (8)	C20—H20A	0.9900
N5—C30	1.381 (10)	C20—H20B	0.9900
N5—H5n	0.86 (3)	C21—H21A	0.9900
N6—C30	1.321 (10)	C21—H21B	0.9900
N6—C34	1.366 (10)	C22—C23	1.410 (11)
C1—C2	1.517 (8)	C22—C27	1.426 (11)
C1—H1A	0.9800	C23—C24	1.356 (11)
C1—H1B	0.9800	C23—H23	0.9500
C1—H1C	0.9800	C24—C25	1.382 (13)
C2—C3	1.529 (8)	C24—H24	0.9500
C2—H2A	0.9900	C25—C26	1.378 (11)
C2—H2B	0.9900	C25—H25	0.9500
C3—C4	1.524 (9)	C26—C27	1.437 (10)
C3—H3A	0.9900	C26—H26	0.9500
C3—H3B	0.9900	C27—C28	1.462 (11)
C4—H4A	0.9900	C28—C29	1.494 (10)
C4—H4B	0.9900	C29—H29A	0.9800
C5—C10	1.394 (10)	C29—H29B	0.9800
C5—C6	1.408 (10)	C29—H29C	0.9800
C6—C7	1.373 (10)	C30—C31	1.385 (11)
C6—H6	0.9500	C31—C32	1.381 (10)
C7—C8	1.374 (11)	C31—H31	0.9500
C7—H7	0.9500	C32—C33	1.379 (12)
C8—C9	1.396 (10)	C32—H32	0.9500
C8—H8	0.9500	C33—C34	1.366 (12)
C9—C10	1.430 (9)	C33—H33	0.9500
C9—H9	0.9500	C34—H34	0.9500
			
O1—Sn1—C4	102.6 (2)	C9—C10—C11	116.3 (6)
O1—Sn1—N3	154.6 (2)	N1—C11—C10	118.4 (6)
C4—Sn1—N3	102.6 (2)	N1—C11—C12	120.7 (7)
O1—Sn1—N1	81.9 (2)	C10—C11—C12	120.9 (6)
C4—Sn1—N1	174.0 (2)	C11—C12—H12A	109.5
N3—Sn1—N1	73.1 (2)	C11—C12—H12B	109.5
O1—Sn1—Cl1	89.21 (15)	H12A—C12—H12B	109.5
C4—Sn1—Cl1	98.35 (19)	C11—C12—H12C	109.5
N3—Sn1—Cl1	84.38 (18)	H12A—C12—H12C	109.5
N1—Sn1—Cl1	85.49 (17)	H12B—C12—H12C	109.5
O1—Sn1—Cl2	95.17 (15)	N3—C13—N2	118.2 (6)
C4—Sn1—Cl2	94.46 (19)	N3—C13—C14	121.6 (7)
N3—Sn1—Cl2	85.67 (17)	N2—C13—C14	120.1 (7)
N1—Sn1—Cl2	81.18 (17)	C15—C14—C13	118.6 (7)
Cl1—Sn1—Cl2	165.23 (6)	C15—C14—H14	120.7
O2—Sn2—C21	103.1 (3)	C13—C14—H14	120.7
O2—Sn2—N6	155.4 (2)	C14—C15—C16	120.5 (7)
C21—Sn2—N6	101.0 (3)	C14—C15—H15	119.7
O2—Sn2—N4	83.8 (2)	C16—C15—H15	119.7
C21—Sn2—N4	171.6 (3)	C17—C16—C15	117.1 (7)
N6—Sn2—N4	72.6 (2)	C17—C16—H16	121.4
O2—Sn2—Cl3	93.4 (2)	C15—C16—H16	121.4
C21—Sn2—Cl3	94.1 (2)	C16—C17—N3	123.1 (8)
N6—Sn2—Cl3	89.3 (2)	C16—C17—H17	118.5
N4—Sn2—Cl3	80.46 (17)	N3—C17—H17	118.5
O2—Sn2—Cl4	88.4 (2)	C19—C18—H18A	109.5
C21—Sn2—Cl4	100.1 (2)	C19—C18—H18B	109.5
N6—Sn2—Cl4	82.92 (19)	H18A—C18—H18B	109.5
N4—Sn2—Cl4	84.77 (17)	C19—C18—H18C	109.5
Cl3—Sn2—Cl4	164.83 (6)	H18A—C18—H18C	109.5
C5—O1—Sn1	128.2 (4)	H18B—C18—H18C	109.5
C22—O2—Sn2	133.1 (5)	C18—C19—C20	114.1 (8)
C11—N1—N2	116.9 (6)	C18—C19—H19A	108.7
C11—N1—Sn1	129.7 (5)	C20—C19—H19A	108.7
N2—N1—Sn1	113.2 (4)	C18—C19—H19B	108.7
C13—N2—N1	117.4 (6)	C20—C19—H19B	108.7
C13—N2—H2N	115 (5)	H19A—C19—H19B	107.6
N1—N2—H2N	124 (5)	C21—C20—C19	112.3 (8)
C13—N3—C17	118.9 (6)	C21—C20—H20A	109.2
C13—N3—Sn1	117.0 (5)	C19—C20—H20A	109.2
C17—N3—Sn1	124.0 (5)	C21—C20—H20B	109.2
C28—N4—N5	117.2 (6)	C19—C20—H20B	109.2
C28—N4—Sn2	128.3 (5)	H20A—C20—H20B	107.9
N5—N4—Sn2	113.3 (4)	C20—C21—Sn2	119.0 (6)
C30—N5—N4	118.8 (6)	C20—C21—H21A	107.6
C30—N5—H5N	123 (5)	Sn2—C21—H21A	107.6
N4—N5—H5N	106 (5)	C20—C21—H21B	107.6
C30—N6—C34	118.9 (7)	Sn2—C21—H21B	107.6
C30—N6—Sn2	118.7 (5)	H21A—C21—H21B	107.0
C34—N6—Sn2	121.8 (5)	O2—C22—C23	115.3 (7)
C2—C1—H1A	109.5	O2—C22—C27	125.1 (7)
C2—C1—H1B	109.5	C23—C22—C27	119.6 (7)
H1A—C1—H1B	109.5	C24—C23—C22	122.0 (8)
C2—C1—H1C	109.5	C24—C23—H23	119.0
H1A—C1—H1C	109.5	C22—C23—H23	119.0
H1B—C1—H1C	109.5	C23—C24—C25	119.9 (8)
C1—C2—C3	111.8 (5)	C23—C24—H24	120.0
C1—C2—H2A	109.2	C25—C24—H24	120.0
C3—C2—H2A	109.2	C24—C25—C26	120.5 (8)
C1—C2—H2B	109.2	C24—C25—H25	119.8
C3—C2—H2B	109.2	C26—C25—H25	119.7
H2A—C2—H2B	107.9	C25—C26—C27	121.8 (8)
C4—C3—C2	114.9 (5)	C25—C26—H26	119.1
C4—C3—H3A	108.5	C27—C26—H26	119.1
C2—C3—H3A	108.5	C22—C27—C26	116.2 (7)
C4—C3—H3B	108.5	C22—C27—C28	125.5 (7)
C2—C3—H3B	108.5	C26—C27—C28	118.0 (7)
H3A—C3—H3B	107.5	N4—C28—C27	121.3 (7)
C3—C4—Sn1	111.6 (4)	N4—C28—C29	120.0 (7)
C3—C4—H4A	109.3	C27—C28—C29	118.7 (6)
Sn1—C4—H4A	109.3	C28—C29—H29A	109.5
C3—C4—H4B	109.3	C28—C29—H29B	109.5
Sn1—C4—H4B	109.3	H29A—C29—H29B	109.5
H4A—C4—H4B	108.0	C28—C29—H29C	109.5
O1—C5—C10	124.1 (6)	H29A—C29—H29C	109.5
O1—C5—C6	116.7 (6)	H29B—C29—H29C	109.5
C10—C5—C6	119.1 (7)	N6—C30—N5	116.2 (7)
C7—C6—C5	121.3 (7)	N6—C30—C31	122.9 (7)
C7—C6—H6	119.4	N5—C30—C31	120.9 (7)
C5—C6—H6	119.4	C32—C31—C30	117.3 (7)
C6—C7—C8	120.9 (7)	C32—C31—H31	121.4
C6—C7—H7	119.6	C30—C31—H31	121.4
C8—C7—H7	119.6	C33—C32—C31	120.7 (7)
C7—C8—C9	119.5 (7)	C33—C32—H32	119.6
C7—C8—H8	120.2	C31—C32—H32	119.6
C9—C8—H8	120.2	C34—C33—C32	118.5 (8)
C8—C9—C10	120.5 (7)	C34—C33—H33	120.8
C8—C9—H9	119.8	C32—C33—H33	120.8
C10—C9—H9	119.8	N6—C34—C33	121.5 (8)
C5—C10—C9	118.8 (7)	N6—C34—H34	119.2
C5—C10—C11	124.9 (6)	C33—C34—H34	119.2
			
C4—Sn1—O1—C5	140.7 (6)	C6—C5—C10—C11	176.4 (7)
N3—Sn1—O1—C5	−45.8 (9)	C8—C9—C10—C5	1.4 (11)
N1—Sn1—O1—C5	−35.4 (6)	C8—C9—C10—C11	−176.5 (7)
Cl1—Sn1—O1—C5	−120.9 (6)	N2—N1—C11—C10	−172.5 (6)
Cl2—Sn1—O1—C5	44.9 (6)	Sn1—N1—C11—C10	13.2 (10)
C21—Sn2—O2—C22	167.9 (8)	N2—N1—C11—C12	4.1 (10)
N6—Sn2—O2—C22	−22.9 (12)	Sn1—N1—C11—C12	−170.3 (5)
N4—Sn2—O2—C22	−7.1 (8)	C5—C10—C11—N1	−18.9 (11)
Cl3—Sn2—O2—C22	72.9 (8)	C9—C10—C11—N1	158.9 (7)
Cl4—Sn2—O2—C22	−92.0 (8)	C5—C10—C11—C12	164.6 (7)
O1—Sn1—N1—C11	8.3 (6)	C9—C10—C11—C12	−17.6 (10)
N3—Sn1—N1—C11	−176.4 (7)	C17—N3—C13—N2	177.3 (7)
Cl1—Sn1—N1—C11	98.1 (7)	Sn1—N3—C13—N2	1.5 (9)
Cl2—Sn1—N1—C11	−88.3 (7)	C17—N3—C13—C14	−2.7 (11)
O1—Sn1—N1—N2	−166.3 (5)	Sn1—N3—C13—C14	−178.4 (6)
Cl1—Sn1—N1—N2	−76.4 (5)	N1—N2—C13—N3	7.2 (10)
Cl2—Sn1—N1—N2	97.2 (5)	N1—N2—C13—C14	−172.9 (7)
C11—N1—N2—C13	172.9 (7)	N3—C13—C14—C15	4.3 (12)
Sn1—N1—N2—C13	−11.8 (8)	N2—C13—C14—C15	−175.6 (7)
C4—Sn1—N3—C13	178.6 (5)	C13—C14—C15—C16	−4.1 (12)
N1—Sn1—N3—C13	−5.7 (5)	C14—C15—C16—C17	2.3 (12)
Cl1—Sn1—N3—C13	81.3 (5)	C15—C16—C17—N3	−0.6 (12)
Cl2—Sn1—N3—C13	−87.8 (5)	C13—N3—C17—C16	0.8 (11)
O1—Sn1—N3—C17	−170.4 (5)	Sn1—N3—C17—C16	176.3 (6)
C4—Sn1—N3—C17	3.1 (6)	C18—C19—C20—C21	−64.4 (11)
N1—Sn1—N3—C17	178.8 (6)	C19—C20—C21—Sn2	−72.9 (9)
Cl1—Sn1—N3—C17	−94.3 (6)	O2—Sn2—C21—C20	86.9 (7)
Cl2—Sn1—N3—C17	96.7 (6)	N6—Sn2—C21—C20	−88.5 (7)
O2—Sn2—N4—C28	18.6 (7)	Cl3—Sn2—C21—C20	−178.7 (6)
N6—Sn2—N4—C28	−168.2 (7)	Cl4—Sn2—C21—C20	−3.9 (7)
Cl3—Sn2—N4—C28	−75.9 (7)	Sn2—O2—C22—C23	177.2 (6)
Cl4—Sn2—N4—C28	107.6 (7)	Sn2—O2—C22—C27	−2.2 (13)
O2—Sn2—N4—N5	−174.0 (5)	O2—C22—C23—C24	−179.1 (8)
N6—Sn2—N4—N5	−0.8 (5)	C27—C22—C23—C24	0.4 (12)
Cl3—Sn2—N4—N5	91.5 (5)	C22—C23—C24—C25	−1.5 (13)
Cl4—Sn2—N4—N5	−85.0 (5)	C23—C24—C25—C26	1.4 (12)
C28—N4—N5—C30	173.8 (7)	C24—C25—C26—C27	−0.2 (12)
Sn2—N4—N5—C30	4.9 (8)	O2—C22—C27—C26	−179.8 (7)
O2—Sn2—N6—C30	13.0 (11)	C23—C22—C27—C26	0.8 (11)
C21—Sn2—N6—C30	−177.8 (6)	O2—C22—C27—C28	6.9 (13)
N4—Sn2—N6—C30	−3.5 (6)	C23—C22—C27—C28	−172.5 (8)
Cl3—Sn2—N6—C30	−83.7 (6)	C25—C26—C27—C22	−0.9 (11)
Cl4—Sn2—N6—C30	83.2 (6)	C25—C26—C27—C28	173.0 (7)
O2—Sn2—N6—C34	−158.4 (6)	N5—N4—C28—C27	172.9 (7)
C21—Sn2—N6—C34	10.9 (7)	Sn2—N4—C28—C27	−20.1 (11)
N4—Sn2—N6—C34	−174.8 (7)	N5—N4—C28—C29	−6.5 (10)
Cl3—Sn2—N6—C34	105.0 (6)	Sn2—N4—C28—C29	160.5 (5)
Cl4—Sn2—N6—C34	−88.1 (6)	C22—C27—C28—N4	5.4 (12)
C1—C2—C3—C4	177.2 (5)	C26—C27—C28—N4	−167.8 (7)
C2—C3—C4—Sn1	−71.1 (6)	C22—C27—C28—C29	−175.2 (7)
O1—Sn1—C4—C3	−1.2 (5)	C26—C27—C28—C29	11.5 (11)
N3—Sn1—C4—C3	−178.3 (4)	C34—N6—C30—N5	178.9 (7)
Cl1—Sn1—C4—C3	−92.2 (4)	Sn2—N6—C30—N5	7.3 (9)
Cl2—Sn1—C4—C3	95.1 (4)	C34—N6—C30—C31	−4.4 (12)
Sn1—O1—C5—C10	42.3 (10)	Sn2—N6—C30—C31	−175.9 (6)
Sn1—O1—C5—C6	−141.8 (5)	N4—N5—C30—N6	−8.1 (10)
O1—C5—C6—C7	−176.0 (6)	N4—N5—C30—C31	175.1 (7)
C10—C5—C6—C7	0.1 (11)	N6—C30—C31—C32	3.2 (12)
C5—C6—C7—C8	1.1 (11)	N5—C30—C31—C32	179.8 (7)
C6—C7—C8—C9	−1.0 (12)	C30—C31—C32—C33	−1.5 (12)
C7—C8—C9—C10	−0.2 (11)	C31—C32—C33—C34	1.1 (13)
O1—C5—C10—C9	174.5 (7)	C30—N6—C34—C33	3.9 (12)
C6—C5—C10—C9	−1.3 (10)	Sn2—N6—C34—C33	175.2 (6)
O1—C5—C10—C11	−7.8 (11)	C32—C33—C34—N6	−2.2 (13)

### Hydrogen-bond geometry (Å, °)

**Table:**

*D*—H···*A*	*D*—H	H···*A*	*D*···*A*	*D*—H···*A*
N2—H2n···Cl4	0.86 (3)	2.47 (4)	3.283 (7)	159 (7)
N5—H5n···Cl2	0.86 (3)	2.48 (5)	3.235 (7)	147 (7)
C15—H15···Cl2^i^	0.95	2.79	3.538 (9)	137
C17—H17···Cl1^ii^	0.95	2.80	3.540 (9)	135
C34—H34···Cl3^iii^	0.95	2.75	3.534 (9)	141

Symmetry codes: (i) *x*−1, *y*, *z*; (ii) *x*, −*y*+2, *z*+1/2; (iii) *x*, −*y*+1, *z*−1/2.
